# Effect of δ-Ferrite Formation and Self-Tempering Behavior on Mechanical Properties of Type 410 Martensitic Stainless Steel Fabricated via Laser Powder Bed Fusion

**DOI:** 10.3390/ma17225614

**Published:** 2024-11-17

**Authors:** Min-Chang Shin, Eun-Joon Chun

**Affiliations:** Department of Materials System Engineering, Pukyong National University, Busan 48513, Republic of Korea

**Keywords:** martensitic 410 stainless steel, laser powder bed fusion (L-PBF), solidification path, δ-ferrite, martensite, tempering

## Abstract

This study explores the formation of δ-ferrite and its self-tempering behavior in the microstructure of Type 410 martensitic stainless steel produced via laser powder bed fusion (L-PBF). The study investigates the correlation between varying energy densities applied during the L-PBF process and the resultant mechanical properties of the as-built specimens. A microstructural analysis shows that with an increase in energy density, the δ-ferrite fraction decreases, while the martensite content increases, leading to changes in tensile strength and elongation. Higher energy densities reduce tensile strength but significantly enhance ductility. The self-tempering effect of martensite in reheated zones, caused by the complex thermal cycling during the L-PBF process, plays a critical role in determining mechanical behavior. These findings provide valuable insights for optimizing the additive manufacturing of martensitic stainless steels to achieve the desired mechanical properties.

## 1. Introduction

Type 410 stainless steel is a prominent type of martensitic stainless steel, characterized by low carbon content (<0.15 wt%C) and chromium content (11.5–13.5 wt%Cr). This alloy is extensively used in key components such as those used in automobiles, maritime applications, and power turbines due to its commendable corrosion resistance, wear resistance, and superior strength [1]. Particularly, parts like compressor blades for power turbines require high dimensional accuracy, and conventional shaping methods like casting, forging, and machining often result in inevitable material loss [2]. Metal additive manufacturing processes, such as laser powder bed fusion (L-PBF), are considered for these manufacturing processes due to their minimal material loss and ability to achieve complex shapes with high dimensional precision [3,4].

From this perspective, recent studies have reported various findings on martensitic stainless steels fabricated using L-PBF. Several studies have examined the effect of post-heat treatment on the microstructure and mechanical properties of Type 420 stainless steel produced by L-PBF [5,6,7]. Tian et al. [5] reported that post-heat treatment increased strength and decreased ductility due to the decomposition of residual austenite. Similarly, Nath et al. [6] also confirmed the decomposition of residual austenite upon post-heat treatment, but in contrast to Tian et al.’s results [5], they reported that both strength and ductility were improved by the tempered degree of martensite. In contrast, Saeidi et al. [7] reported that the austenite fraction increased following post-heat treatment, leading to an increase in the transformation-induced plasticity effect, which resulted in higher strength and ductility. This improvement in mechanical properties due to reverted austenite transformation has also been reported for the heat treatment of L-PBF-fabricated PH 17-4 alloy [8]. Li et al. [9] investigated the effect of post-heat treatment on the mechanical properties of PH 17-4 alloy prepared by L-PBF, reporting that austenite transforms into martensite upon post-heat treatment and that mechanical properties can be enhanced by controlling the aging temperature. In addition, Benoit et al. [10] and Moniruzzaman et al. [11] reviewed the effect of post-heat treatment on the mechanical properties of PH13-8Mo alloy prepared by L-PBF and WAAM, highlighting the differences due to process characteristics. Most of these studies focus on the changes in microstructure and mechanical properties due to post-heat treatment of additively manufactured parts. Since L-PBF involves repeated melting and solidification, heat treatment behavior based on microstructural differences due to varied laser process parameters has been examined in previous studies. Therefore, it is considered that changes in microstructure and mechanical properties due to post-heat treatment are directly influenced by the microstructure of the as-built material, necessitating a prior examination of the microstructure control of as-built materials. However, research on martensitic stainless steel parts fabricated by additive manufacturing is very limited.

In this study, we fundamentally evaluate the correlation between the microstructure and mechanical properties of as-built Type 410 stainless steel fabricated under various energy density conditions, particularly focusing on solidification and reheating phenomena in the melt pool and heat-affected zones. Based on these investigations, the authors elucidate the key microstructural factors necessary to achieve a balance of strength and ductility in the mechanical properties in the as-built condition.

## 2. Materials and Methods

### 2.1. Materials

In this study, Type 410 stainless steel powder provided by HANA AMT was used. This powder has a spherical morphology with an average particle size (D_0.5_) of 28 μm and a size distribution ranging from 15 to 53 μm. The chemical composition of the powder is presented in Table 1.

### 2.2. L-PBF Additive Manufacturing

**Figure 1:**
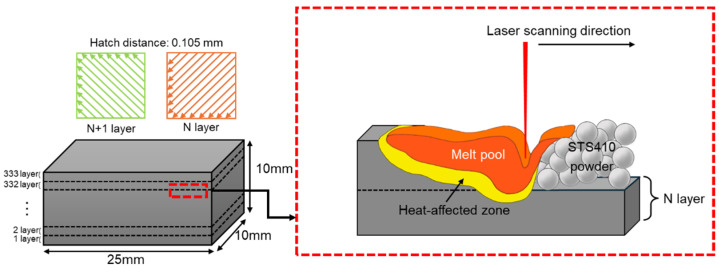
A schematic description of the sample geometry, laser scanning strategy, and L-PBF process used.

The additive manufacturing process utilized a GE Additive Concept Laser M1 Cusing machine. Figure 1 illustrates a schematic diagram of the L-PBF additive manufacturing process. A total of 333 layers were deposited to fabricate an as-built 410 stainless steel specimen measuring 25 × 10 × 10 mm^3^. The laser scanning applied a linear pattern with a 45° inclination angle and a hatch distance (h) of 0.105 mm. The laser scanning direction for each subsequent layer was perpendicular to the previous layer, with the layer thickness (t) set at 30 μm. To investigate the behavior of microstructures according to the additive manufacturing process parameters, a total of 11 conditions were applied within a scan speed (v) range of 238–1587 mm/s and an energy density ($E_{v}$) range of 50–400 J/mm^3^. Here, the energy density calculation is based on Equation (1) below [12]. The details of the conditions are summarized in Table 2.

(1) $$E_{v}=\frac{P}{v\times h\times t}$$

### 2.3. Microstructural Analysis and Mechanical Properties

**Figure 2:**
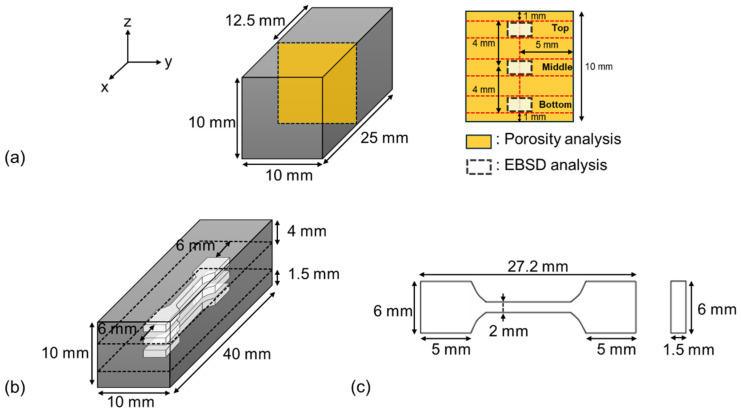
Schematics of (**a**) observation position for microstructure analysis, (**b**) extraction position of tensile test specimens, and (**c**) dimensions of tensile test specimens.

The microstructural characteristics and mechanical properties of 11 types of as-built materials (Conditions A-K) were evaluated without any post-heat treatment. The observation of the microstructure in the additive manufacturing parts was carried out on the yz-plane, which was sectioned from the center of the specimen in the x-axis direction, as shown in Figure 2a. The porosity of the additive manufacturing parts was analyzed using an optical microscope (OM; Leica DM IRM, Leica Microsystems (Wetzlar, Germany)) and ImageJ software. The microstructure analysis utilized Electron Backscatter Diffraction (EBSD; Velocity super, EDAX (Mahwah, NJ 07430, USA)). To minimize errors in the EBSD analysis, we excluded regions with a confidence index (CI) < 0.1 in the inverse pole figure (IPF), phase, and kernel average misorientation (KAM) mapping. To distinguish δ-ferrite and martensite in the EBSD analysis, the authors followed the same technical procedures used in references [13,14,15], based on image quality (IQ). Specifically, a threshold value was determined from the characteristic shape of the chart, which displayed two maxima in the IQ data. According to the chart, the lower part was identified as martensite, while the other parts were identified as ferrite. The mechanical properties of the additive manufacturing parts were assessed through room-temperature tensile tests. The locations for the tensile test specimens were taken from the top, middle, and bottom of the additive manufacturing parts, as shown in Figure 2b, and were prepared according to the KS B 0801 standard which is linked with ISO 6892-1:2019 (Figure 2c). A universal testing machine (UNITECH-T, R&B (Daejeon, Republic of Korea)) was used for the tensile tests, applying a load of 10 kN and a strain rate of 0.2 mm/min. Room-temperature tensile tests were conducted three times for each additive manufacturing condition, and the average values of total elongation and ultimate tensile strength obtained from each test were used. To highlight the effect of energy density on the microstructural characteristics and mechanical properties of L-PBFed Type 410 stainless steel, the results under three representative conditions (conditions “C”, “F”, and “J”) are introduced and discussed in the following section.

## 3. Results and Discussion

### 3.1. Macrostructure of As-Built Type 410 Stainless Steels

**Figure 3:**
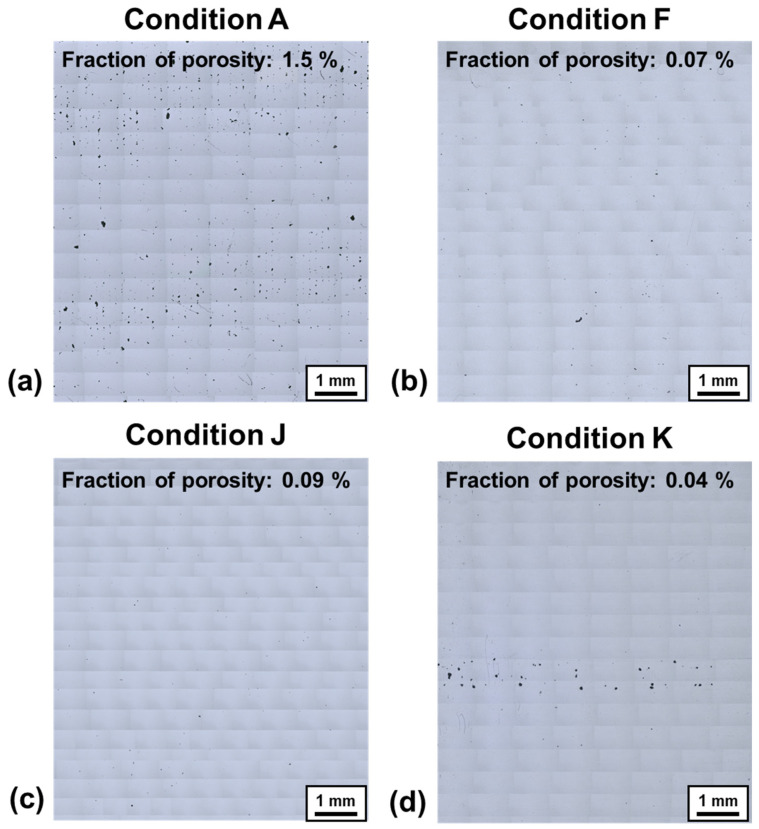
The OM images and fraction of porosity under L-PBF conditions of (**a**) “A”, (**b**) “F”, (**c**) “J”, and (**d**) “K”.

Porosity is one of the most common defects that occur during L-PBF, and can lead to stress concentration during deformation and subsequently cause cracks. In this section, the porosity rates of additive manufacturing parts under 11 different laser scanning conditions were fundamentally examined. Figure 3 shows the OM images and the porosity rates for conditions “A”, “F”, “J”, and “K”, respectively. The porosity rate for Condition “A” (Figure 3a) was assessed at 1.5%, while the porosity rates for conditions “F”, “J”, and “K” were, respectively, 0.07%, 0.09%, and 0.04%. It is known that the porosity rate during L-PBF can be influenced by the laser scanning conditions and the properties of the molten metal. The high porosity rate (1.5%) in condition “A” is thought to result from low power (180 W) and fast scanning speed (1000 mm/s), which leads to incomplete melting and high viscosity of the molten metal [16,17]. Conversely, under the high-power (350 W) condition, the average porosity rate was 0.06%, which indicates a consistent level regardless of the scanning speed.

### 3.2. Variation in Mechanical Properties with Different Energy Density Conditions

**Figure 4:**
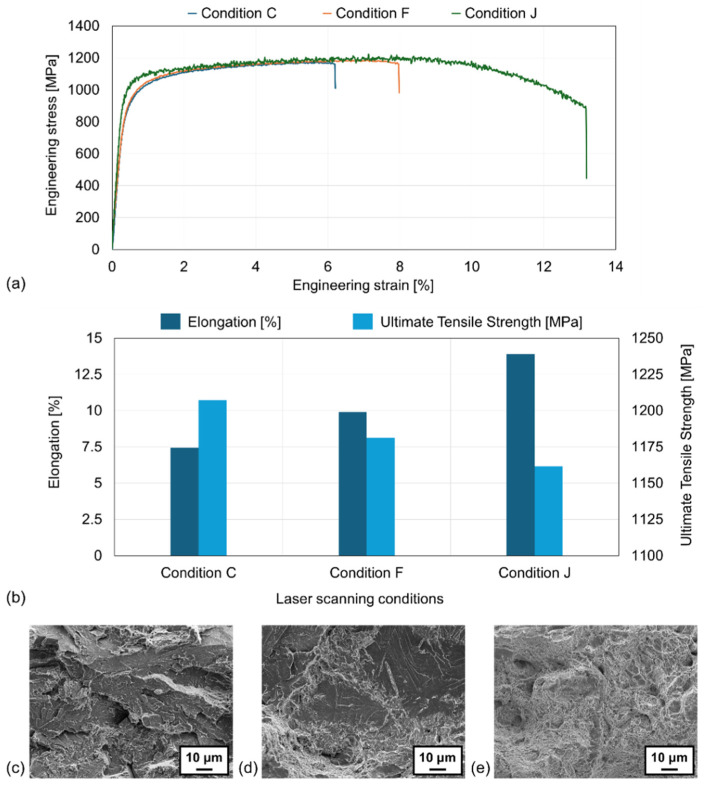
(**a**) Representative stress–strain curve obtained from tensile tests. (**b**) Correlation between laser scanning condition and mechanical properties. SEM fractography for tensile tested specimens under conditions (**c**) “C”, (**d**) “F”, and (**e**) “J”.

Tensile tests were conducted on the as-built L-PBFed specimens, and Figure 4a displays the representative tensile test results for three conditions with significant differences in energy density (conditions “C”, “F”, and “J”). Figure 4b compares the average values of tensile strength and elongation, measured three times for each condition. For condition “C” (energy density: 65 J/mm^3^), the average tensile strength was 1207 MPa, and the elongation was 7.4%; for condition “F” (energy density: 80 J/mm^3^), they were 1181 MPa and 9.9%; and for condition “J” (energy density: 200 J/mm^3^), they were 1162 MPa and 13.9%. As the energy density increased, the tensile strength decreased by approximately 3.7%, while the elongation increased by about 87.8%. Figure 4c–e show the representative fractographic observations for conditions “C”, “F”, and “J”. Conditions “C” (Figure 4c) and “F” (Figure 4d) exhibited a mixture of brittle cleavage fractures and ductile dimple fractures, whereas Condition “J” (Figure 4e) predominantly showed typical ductile fractures with micro-dimples. Thus, the observations of the fracture surfaces also accurately reflected the results of the tensile tests.

### 3.3. Relationship Between Mechanical Properties and Microstructural Characteristics

In this section, we aim to elucidate the reasons behind the differences in mechanical properties shown in Figure 4b by conducting a detailed analysis of the microstructure of Type 410 stainless steel fabricated via L-PBF under different laser scanning conditions. Particularly, this study focuses on examining the solidification path, phase transformation behavior, and behavior of reheated zones’ formation due to differentiated energy density values.

#### 3.3.1. Behavior of Residual δ-Ferrite

The Type 410 stainless steel used is known to have a solidification path and phase transformation behavior in an equilibrium state from liquid (L) to a liquid plus ferrite primary phase (L + Fp), then to a ferrite primary phase (Fp), to austenite (A), mixed martensite and austenite (M + A), and finally, to martensite (M) [10,18]. However, under non-equilibrium solidification and phase transformation conditions such as those in L-PBF, the pathway changes to L → L + Fp + (A + Fe) → Fp + A + Fe → A + Fe → M + Fe [19], thus suppressing the complete transformation to austenite and ultimately resulting in a mixed microstructure of martensite and eutectic δ-ferrite (Fe).

**Figure 5:**
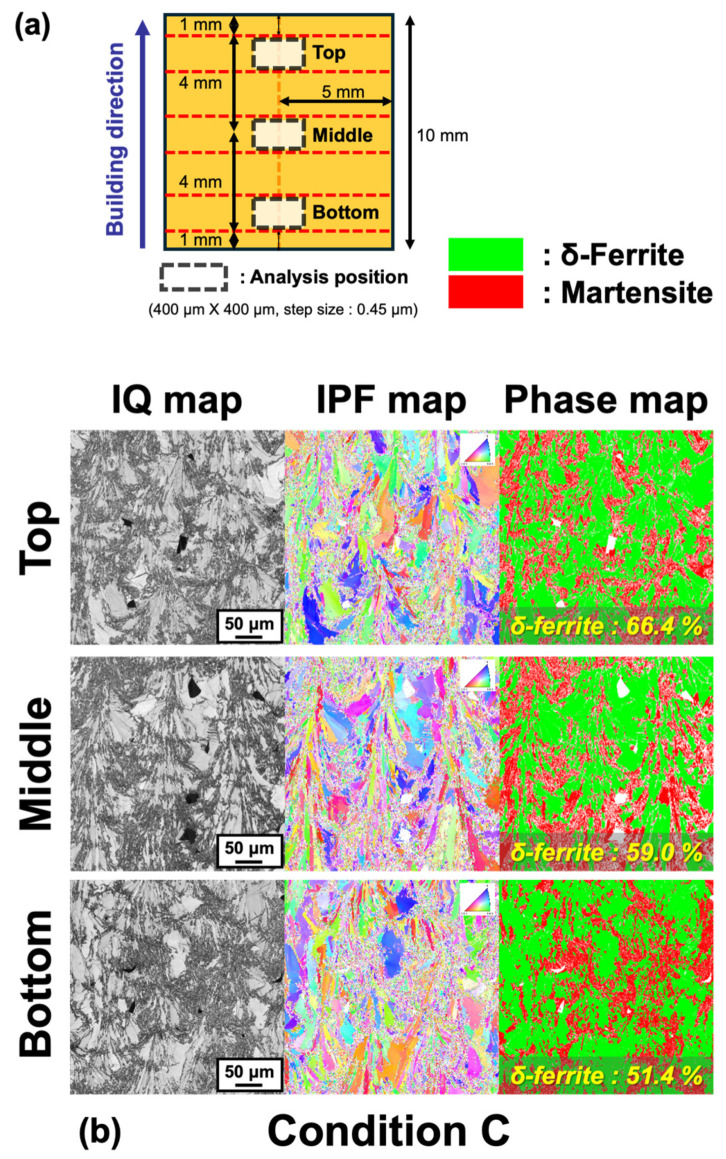

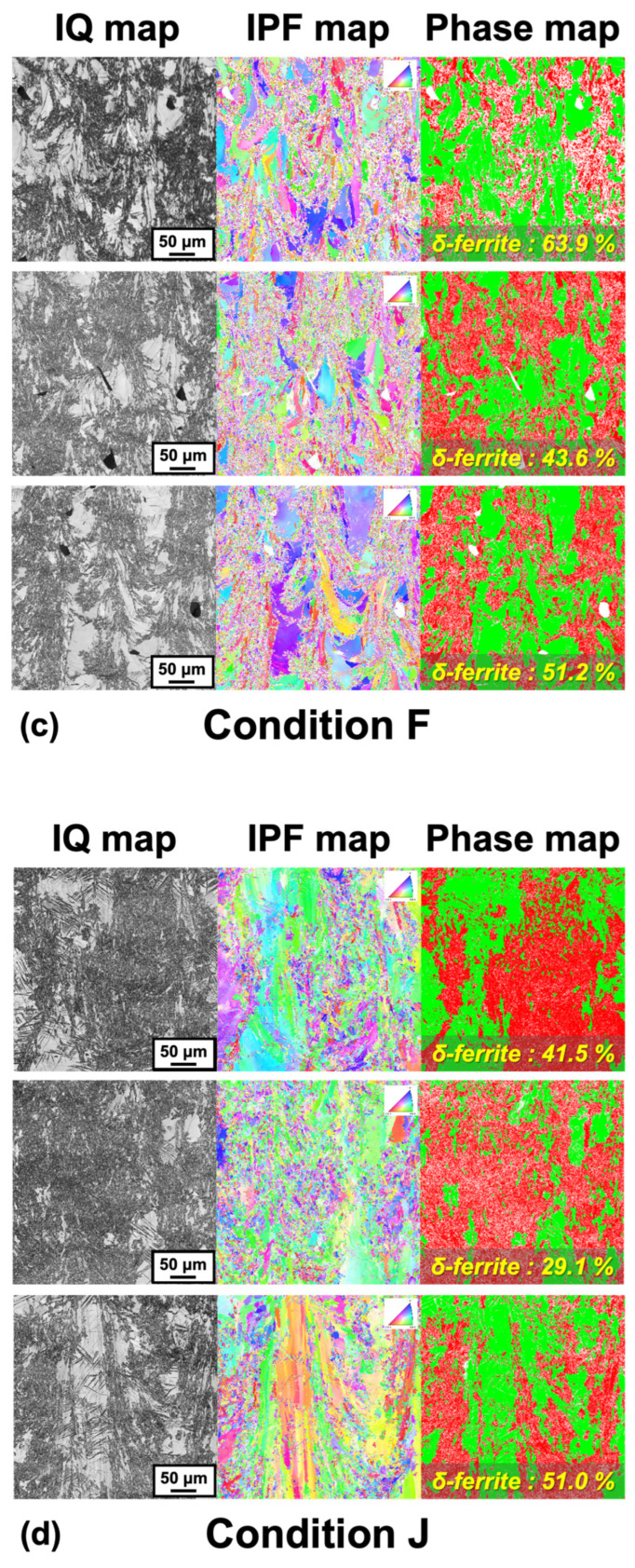
(**a**) Schematic description of analysis points for EBSD. Results of EBSD analysis under conditions (**b**) “C”, (**c**) “F”, and (**d**) “J”, respectively.

**Figure 6:**
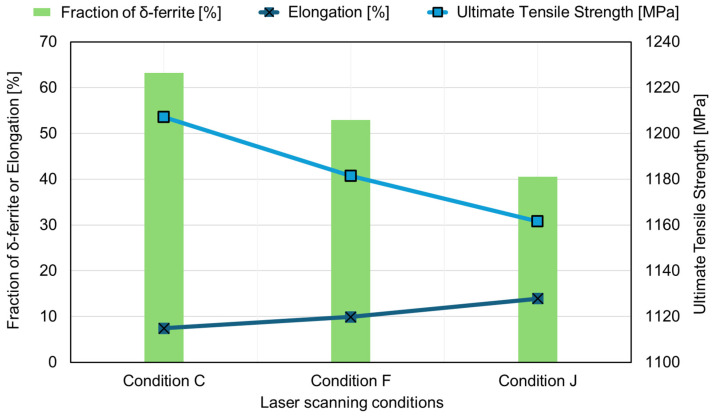
Relationship between energy density and the mechanical properties of L-PBFed Type 410 stainless steels.

Figure 5a shows the location where EBSD analysis was performed on the L-PBFed specimen, and Figure 5b–d represent the typical EBSD analysis results for conditions “C”, “F”, and “J”, respectively. Considering that the thermal history and accumulated heat can vary locally, EBSD analysis was conducted at three positions (top, middle, bottom) along the z-direction of layering. As indicated by the IQ and phase map, the microstructure of the manufacturing parts under all conditions was confirmed to consist of a dual phase of δ-ferrite and martensite. The fraction of δ-ferrite was evaluated as follows: condition “C” (top: 66.4%; middle: 59.0%; bottom: 51.4%; average: 58.9%) > condition “F” (top: 63.9%; middle: 43.6%; bottom: 51.2%; average: 52.9%) > condition “J” (top: 41.5%; middle: 29.1%; bottom: 51.0%; average: 40.5%). Thus, the energy density conditions used in this study were assessed to influence the ratio of δ-ferrite and martensite in the L-PBF manufacturing parts, particularly finding that as the energy density increased, the fraction of δ-ferrite decreased and that of martensite increased. Such differences in phase proportions were anticipated to influence the results of the tensile tests, as shown in Figure 4, and the correlation between the δ-ferrite fraction and tensile test outcomes was examined. Figure 6 displays these results. It was clearly observed that despite the decrease in the fraction of the softer phase, δ-ferrite (with an increase in the harder martensite fraction), as the energy density increased from condition “C” to “F” to “J”, the tensile strength decreased, while elongation increased. Therefore, these findings suggest that the mechanical property behavior of as-built Type 410 stainless steel according to energy density cannot be solely considered from the solidification path and phase proportions, but requires examination from other microstructural influences.

#### 3.3.2. Behavior of Tempered Martensite in Reheated Zone

**Figure 7:**
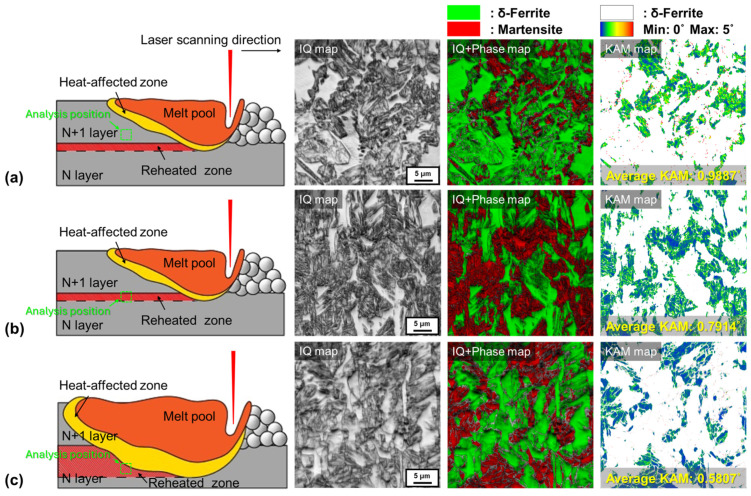
Results of EBSD analysis. (**a**) Melt zone of condition “C” and reheated zones of conditions (**b**) “C” and (**c**) “J”.

From a welding metallurgy perspective, it has been reported that the heat-affected zone (HAZ) of Type 410 stainless steel exhibits a softening phenomenon relative to the base metal due to the tempering of martensite [18]. In this study, the repeated reheating during the L-PBF process leads to the formation of reheated zones, which exhibit more complex tempering behaviors than the HAZ in welding, and are expected to directly affect the mechanical properties. Therefore, using EBSD analysis (KAM), we intend to investigate the mechanical property differences shown in Figure 6 by examining the tempering behaviors of martensite in the reheated zones of conditions “C” and “J”, which have significant differences in their δ-ferrite and martensite fractions, as depicted in Figure 5. The analysis was conducted in the middle of the additive manufacturing parts (Figure 5a) at the boundary of the melt pool in the reheated zones of martensite, and for comparison, the melt zone martensite for condition “C” was also included. Figure 7 shows the results of the EBSD and KAM analyses. Specifically, KAM analysis was conducted only on the martensite phase, as identified in the phase maps and displayed in Figure 7. Figure 7a shows the results for the melt zone of condition “C”, while Figure 7b,c show the results for the reheated zones of conditions “C” and “J”, respectively. In all KAM analysis images, a high dislocation density was observed in the martensite. The average KAM values, which can be regarded as an indicator of tempering degree, were as follows: melt zone of condition “C” (0.9887°) > reheated zone of condition “C” (0.7914°) > reheated zone of condition “J” (0.5807°). The lower KAM values in the reheated zones imply a lower dislocation density compared to the martensite formed in the melt zones, suggesting that the reheating phenomenon during the additive manufacturing process tempers the martensite formed in the melt zones. Thus, the changes in mechanical properties according to the δ-ferrite and martensite composition ratios shown in Figure 6 can be explained as follows: In condition “J”, despite a higher martensite fraction (59.5%) compared to condition “C” (martensite: 41.1%), a decrease in tensile strength and an increase in elongation occur because the higher energy density leads to relatively extensive reheated zones, and tempering of martensite in these reheated zones occurs at a higher level compared to the reheated zones in condition “C”.

#### 3.3.3. Comparison of Mechanical Properties of Type 410 Stainless Steel Produced by L-PBF in As-Built Condition and Other Processes

**Figure 8:**
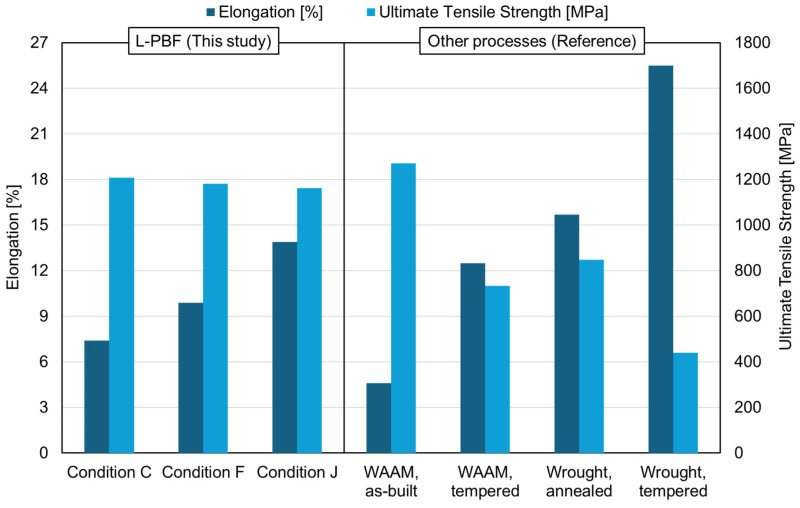
Summary of mechanical properties evaluated in this study and other processes (as-built WAAM [20], tempered WAAM [20], annealed wrought [21], tempered wrought [22]) of Type 410 stainless steels.

As explained in the previous section, the mechanical properties of L-PBFed Type 410 stainless steel are changed with varying energy density in the as-built state. In particular, its strength (1162 MPa) and ductility (13.9%) are the most balanced properties for condition “J” compared with other L-PBF conditions. Therefore, the newly achieved balanced mechanical properties were compared with those fabricated by other processes. Figure 8 summarizes the comparison results. Compared with the properties of condition “J”, the results of Type 410 stainless steel fabricated by WAAM [20] exhibit a considerably low level of ductility (4.6%) while showing a similar level of strength (1270 MPa) under the as-built state. After post-WAAM heat treatment, ductility is significantly improved (from 4.6% to 12.5%), while strength shows a notable decrease (from 1270 MPa to 734 MPa), resulting in more balanced properties. However, the levels of strength and ductility are lower than those of L-PBF (condition “J”). Figure 8 also displays the properties of wrought Type 410 stainless steel in both the as-annealed [21] and tempered [22] states. In the case of the annealed state, a balance of properties was achieved (848 MPa and 15.7%), offering a similar balance to that of L-PBFed (condition “J”) as-built materials. However, tempered wrought Type 410 stainless steel shows extremely unbalanced properties (440 MPa and 25.5%). To summarize, the balanced mechanical properties achieved in L-PBFed and as-built martensitic stainless steel are similar to those of annealed wrought Type 410 stainless steel and are superior to those obtained by WAAM alone. Consequently, this study provides meaningful insights into as-built L-PBFed Type 410 materials.

## 4. Conclusions

This study provides an in-depth evaluation of the influence of δ-ferrite formation and self-tempering behavior on the mechanical properties of as-built Type 410 martensitic stainless steel fabricated via laser powder bed fusion (L-PBF). The conclusions are summarized as follows:

The microstructural analysis of as-built Type 410 stainless steel specimens demonstrated that increasing the energy density during the L-PBF process leads to a reduction in the δ-ferrite fraction (from 58.9% to 40.5%) and an increase in martensite content (from 41.1% to 59.5%). These microstructural variations result in a decrease in tensile strength (from 1207 to 1162 MPa) and a substantial increase in elongation (from 7.3 to 13.9%). The mechanical properties of as-built Type 410 stainless steel are thus not solely dependent on the ferrite fraction. Instead, the observed unexpected results are attributed to variations in martensite tempering behavior within the reheated zones.

Specifically, conditions with higher energy density exhibit relatively wide reheated zones with a higher fraction of tempered martensite, which directly influences the mechanical properties, increasing ductility by 87.8% and decreasing strength by only 3.7%. These results highlight the importance of controlling energy density and the resulting microstructure (particularly the tempering of martensite) to achieve balanced mechanical performance (strength and ductility) in L-PBFed Type 410 stainless steel. Future studies should focus on optimizing processing conditions to enhance both strength and ductility for industrial applications by comparing various AM processes.

## Figures and Tables

**Table 1:** Chemical composition of Type 410 stainless steel powder used (mass%).

Fe	Cr	Mn	Ni	Si	C
Bal.	12.56	0.84	0.35	0.24	0.106

**Table 2:** The specific laser scanning conditions for the L-PBF process.

Conditions	Defocus Distance (mm)	Beam Size (mm)	Laser Power (W)	Scan Speed (mm/s)	Energy Density (J/mm^3^)
A	+3	0.096	180	1000	50
B			350	1587	60
C				1465	65
D				1361	70
E				1270	75
F				1190	80
G				1120	85
H				1058	90
I				635	150
J				476	200
K				238	400

## Data Availability

The data presented in this study are available on request from the corresponding author.
